# Simultaneous Detection of Displacement, Rotation Angle, and Contact Pressure Using Sandpaper Molded Elastomer Based Triple Electrode Sensor

**DOI:** 10.3390/s17092040

**Published:** 2017-09-06

**Authors:** Eunsuk Choi, Onejae Sul, Seung-Beck Lee

**Affiliations:** 1 Department of Electronic Engineering, Hanyang University, 222 Wangsimni-ro, Seongdong-gu, Seoul 133-791, Korea; silver77@hanyang.ac.kr; 2 Institute of Nano Science and Technology, Hanyang University, 222 Wangsimni-ro, Seongdong-gu, Seoul 133-791, Korea; ojsul@hanyang.ac.kr

**Keywords:** motion sensor, displacement sensor, rotation angle sensor, pressure sensor, sandpaper molded PDMS

## Abstract

In this article, we report on a flexible sensor based on a sandpaper molded elastomer that simultaneously detects planar displacement, rotation angle, and vertical contact pressure. When displacement, rotation, and contact pressure are applied, the contact area between the translating top elastomer electrode and the stationary three bottom electrodes change characteristically depending on the movement, making it possible to distinguish between them. The sandpaper molded undulating surface of the elastomer reduces friction at the contact allowing the sensor not to affect the movement during measurement. The sensor showed a 0.25 mm^−1^ displacement sensitivity with a ±33 μm accuracy, a 0.027 degree^−1^ of rotation sensitivity with ~0.95 degree accuracy, and a 4.96 kP^−1^ of pressure sensitivity. For possible application to joint movement detection, we demonstrated that our sensor effectively detected the up-and-down motion of a human forefinger and the bending and straightening motion of a human arm.

## 1. Introduction

Recently, many groups have reported on various motion sensors for possible real-time personal health monitoring, human-robot interfaces, and industrial robot applications [1,2,3,4,5,6,7,8]. Motion sensing is achieved by attaching sensors at, or near, joints in robots or humans and monitoring their displacement or rotational angle. When monitored in parallel to the motion, the bending of a joint causes points on both sides to become displaced and the displacement sensor measures the change in distance. When monitored normally to the bending plane, limbs connected to the joint counter rotate and the rotation sensor measures the angular change made at the joint. While there are human joints that only have a single axis of rotation such as the elbow, whose motion can be monitored by a single sensor, many joints—such as the finger socket, wrist, and shoulder joints—have multiple axes of rotation and therefore requires simultaneous measurement of several displacement or rotation angle sensors to monitor their motion accurately [8,9]. For this purpose, there have been several reports of sensors that utilize capacitive and optical sensing schemes capable of monitoring multi-axis movements. These sensors are capable of simultaneously sensing displacement, with tens of microns’ sensitivity, and rotation angles, with sub-degree accuracy [10,11,12,13]. Although these sensors demonstrate high sensitivity and resolution, they have complex structures and considering that human joint movements are the result of muscle contractions, the provided sensing accuracies are unnecessarily high [14].

**Figure 1:**
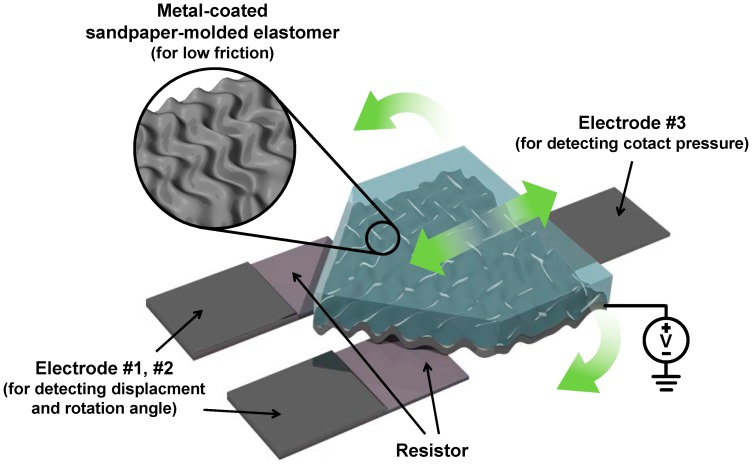
A schematic diagram of the triple contact electrode sensor based on sandpaper-molded elastomer.

In this study, we developed a simple sandpaper molded elastomer-based triple electrode sensor that can discriminate simultaneously between displacement, rotation angle, and applied tactile pressure (see Figure 1). When displacement and rotation were applied, they were detected by the difference in the resistance change, coming from the change in contact area, between two separate electrodes (electrode no. 1 and no. 2) both making contact with the metal-coated sandpaper-molded elastomer. The undulating surface structure provided by the sandpaper structural mold reduces the friction between the contacting electrodes considerably, allowing the sensor to operate without affecting the measured output. When external pressure was applied, all three of the electrode contact areas increased, making contact pressure detection possible. Our sensor showed a minimum 0.25 mm^−1^ of displacement sensitivity with a ±33 μm accuracy, a minimum 0.027 degree^−1^ of rotation sensitivity with ~0.95 degree of angular accuracy, and a 4.96 kP^−1^ of pressure sensitivity. We also show that it is possible to monitor human joint movements using our sensor by demonstrating the detection of the up-and-down tilting motion of a human forefinger and the bending and straitening motion of a human elbow. With further developments, the sandpaper molded elastomer sensor may become a low cost, facile, and reliable alternative to the highly cumbersome and high cost sensors that are currently used.

## 2. Sandpaper Molded PDMS

**Figure 2:**
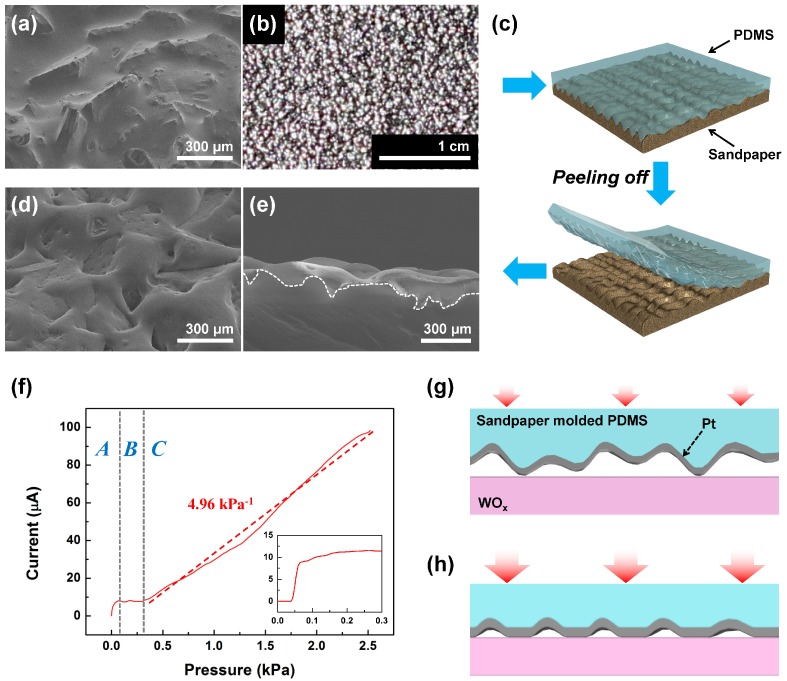
The sandpaper molded PDMS (SMP); (**a**) The SEM image and (**b**) the optical image of a sandpaper with a grit size of 80 (CAMI grit designation); (**c**) the molding process of SMP; (**d**) the oblique angle and (**e**) cross-sectional SEM images of SMP, where the white dashed line shows the cross-sectional boundary; (**f**) the pressure dependent output current of a basic tactile sensor based on the SMP at an applied bias of 10 mV, where the inset shows the expanded output characteristics of another sensor, and the schematic illustrations of SMP tactile sensor in region (**g**) A and (**h**) C in Figure 2f.

We used a sandpaper structural master for its simplicity, standardization, and low cost. Sandpapers show high surface roughness, over tens of microns’ height difference (see Figure 2a), with uniform distribution of roughness over a wide area (see Figure 2b) which makes them suitable as a structural master for the type of sensor we are developing. The sandpaper we used has a grit size of 80 (CAMI grit designation), silicon carbide particles with about 190 μm average size and 10^3^ cm^−2^ density, as shown in Figure 2b. To fabricate the active sensor film, we transferred the surface pattern of the sandpaper by poly(dimetlysiloxane) (PDMS) molding (see Figure 2c). The PDMS was poured on the sandpaper and cured, and then was peeled off. The 2-mm-thick sandpaper molded PDMS (SMP) shows an irregular undulating surface structure, as shown in Figure 2d. The cross-section SEM image in Figure 2e shows the surface roughness profile of the SMP which was closely mirroring that of the sandpaper master. The SMP was highly deformable with the deformability controlled by the PDMS mixture ratio of 10:1 (base: curing agent) giving a Young’s modulus of 750 kPa.

A basic tactile sensor based on the SMP was fabricated by sputtering 30 nm of Pt on the SMP surface and then placing it on a PET film which has a 80 nm layer of sputtered WO_x_. Pt was used for its adhesion to PDMS, and as the material for the contact resistor, WO_x_ was used for its resistivity and also to reduce adhesion with the Pt. Figure 2f shows the pressure dependent current of the SMP tactile sensor at an applied bias of 10 mV. In region A, pressure range under 100 Pa, the current was dramatically increased by the initial pressure. Due to the irregular height of the ridges, the SMP microstructure made sequential electrical contacts to the WO_x_ layer with minimum external pressure, which increased the output current dramatically (see illustration shown in Figure 2g). Then, in region B, little current change was observed due to the applied pressure not being high enough to deform the undulating SMP surface structure. This initial pressure dependent characteristic in region A and B was also observed in other sensors we fabricated, as shown in the inset of Figure 2f and can also be seen in previously reported tactile sensors that used microstructures as structural masters [15,16,17,18,19,20]. Therefore, we may consider the sensor output in regions A and B as the contact pressure threshold, meaning that it can be used to detect initial contact. When applied pressure exceeds 300 Pa in region C, wider portions of the undulated SMP surface deforms from the pressure, and the contact area between the upper SMP structure and the lower electrode increases, as illustrated in Figure 2h. Thus, the sensor current increases as the contact area increases. The pressure sensitivity in region C was measured at 4.96 kPa^−1^ which was comparable to the pressure sensitivity of reported tactile pressure sensors that relied on microstructure deformation as their sensing mechanism [17,18,19,20,21,22]. Above 2.5 kPa, the output current showed saturation due to the undulated SMP surface being almost completely flattened. Our sensor shows nonlinear operation in regions A and B due to its undulating surface. To prevent nonlinear operation at initial pressure, a preload of 500 Pa was applied for actual sensor operation.

## 3. Dual Electrode SMP Sensor

### 3.1. Sensor Design

**Figure 3:**
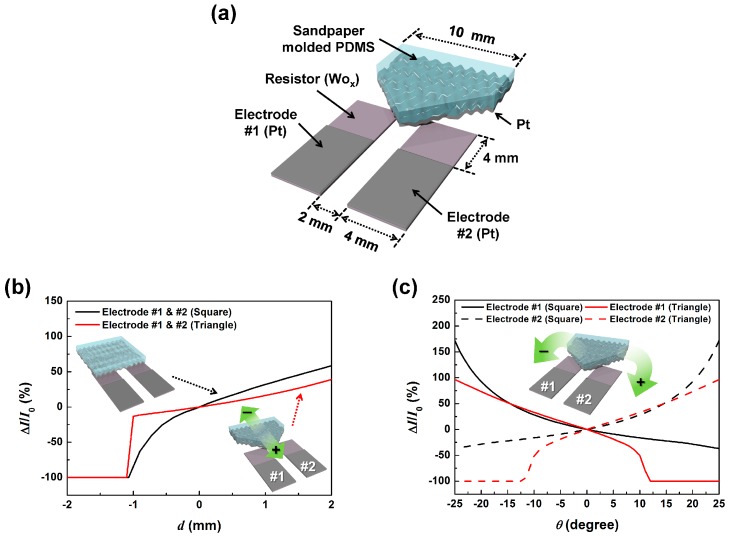
The dual electrode SMP (dSMP) sensor; (**a**) a schematic diagram of the dSMP sensor; (**b**) the simulation results on the current change ratios (Δ*I*/*I*_0_) of the lower electrodes of the dSMP sensor depending on the triangular (black) and rectangular (red) shape of SMP by applied displacement, and (**c**) the simulation results on Δ*I*/*I*_0_ of electrode no. 1 (solid) and no. 2 (dash) depending on the triangular (black) and rectangular (red) SMP shape. Insets show the schematic illustration of triangular and rectangular shape SMP, and the green arrows shows the movement direction.

Previously reported tactile sensors using microstructure deformation did demonstrate that they can sense not only pressure but also shear and torsion, which may be used to determine displacement and rotation angle [15,16,17,18]. However, this was just a demonstration of the fact that the various external stimuli produce changes in the sensor output, not that the sensor was operating simultaneously as a pressure, shear, and torsion sensor. More fundamentally, due to the sensor structure and contact configuration, these sensors are not able to discriminate between the external contact pressure, shear, and torsional stimuli. To make a distinction between the external stimuli, we designed a dual electrode SMP (dSMP) sensor structure as shown in Figure 3a. This sensor is composed of two lower 80 nm thick WO_x_ resistors with 80 nm thick Pt electrodes and a triangular SMP making contact. The distinction between displacement and rotation angle was made simply by comparing the output currents from the two electrodes. When displacement or rotation is applied, it changes the contact area, and therefore the resistance, between the SMP layer and the two electrodes. Due to the arrangement of the electrodes, the resistance changes in unison during one-dimensional displacement motion and opposite to each other during rotational motion.

For high linearity and sensitivity, we designed the SMP electrode to have a triangular shape, as shown in Figure 3a. This triangular shape was chosen based on simulations of the applied displacement dependent current change ratios Δ*I*/*I*_0_ (where *I*_0_ is the initial current at the zero position and Δ*I* is the difference in measured current to *I*_0_) of the dSMP sensor. Figure 3b shows the simulation result (Comsol multiphysic 5.2) when displacement was applied from left (minus) to right (plus), as shown in the inset illustration. With the displacement, the contact area between the SMP and the bottom electrodes change and, regardless of the electrode shape, the change is the same for the two electrodes. However, the change was more linear and had a higher sensitivity to movement for the triangle shaped SMP than the rectangular one. Figure 3c shows the simulation result on Δ*I*/*I*_0_ of the dSMP sensor depending on applied rotation. We set the clockwise rotation as the positive angle and the counterclockwise rotation as the negative rotation angle, as shown in the inset illustration. The Δ*I*/*I*_0_ of the two electrodes were observed to be opposite to each other depending on applied rotation. Overall, the triangular shape SMP shows higher angular sensitivity to the rectangular shaped SMP. However, a dramatic decrease of the output current was seen in the range of about 10 degrees, which is due to the SMP losing contact with the bottom electrodes similarly to the displacement sensing mode. Considering that there is no other SMP motion that can cause only one of the contact electrodes to give an output signal, the current change of the other in-contact electrode may be used to calculate the rotation angle beyond 10 degrees of rotation. The simulations show that the dSMP sensor has the ability to discriminate between displacement and rotation, and that the dSMP with the triangular shape SMP contact had a more linear response and a higher sensitivity to that with a rectangular shape.

### 3.2. Measurement Error Analysis

**Figure 4:**
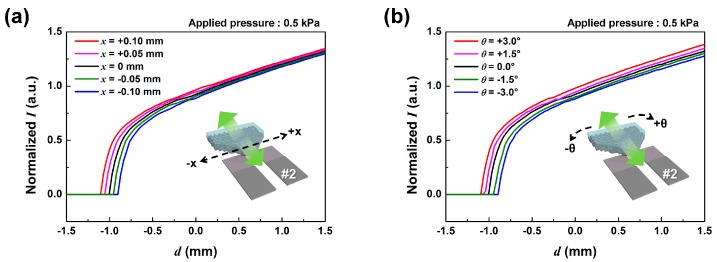

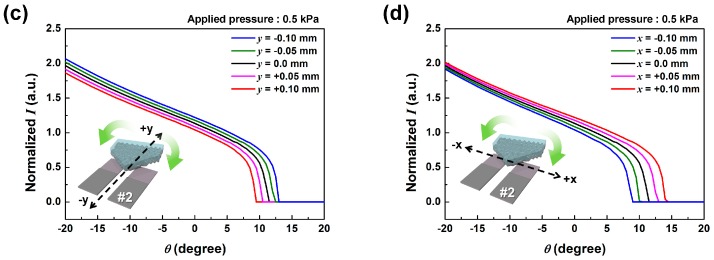
The effect of out-of-axis movements of SMP on sensor detection; the simulated displacement sensing error of electrode no. 2 depending on (**a**) the lateral displacements of ±100 μm with 50 μm interval, and (**b**) the rotation angles of ±3 degrees with a 1.5 degree interval, and the simulated rotation angle sensing error of electrode no. 2 depending on ±100 μm displacement error with 50 μm intervals for (**c**) the parallel and (**d**) lateral movements. The insets show the schematic illustrations of dSMP sensor movement, the green arrows show the movement direction, and the black dash lines show the axes of the initial position error.

The top SMP needs to move back and forth and also rotate to detect displacement and rotation angle simultaneously, meaning that it requires two degrees of freedom: one translational and one rotational. This puts limitations on the accuracy of the sensor, since the SMP movement cannot be limited to a single axis. Therefore, possible measurement error depending on out-of-axis movements should be considered. Figure 4 shows the simulated effect of the out-of-axis movements of SMP on sensor detection results. We can see from Figure 4a,b that a lateral displacement of ±100 μm and a rotation of ±3 degrees would cause a displacement current measurement error of ±5%. Also from Figure 4c,d, we see that the parallel and lateral displacement error of both ±100 μm would cause the detected rotation angle to have an average ±10% error. This shows that it becomes necessary to limit the out-of-axis movement of the top SMP as much as possible while maintaining low contact friction between SMP and the contact electrodes. For this purpose, we have utilized an elastic casing to provide a vertical pressure point to act as a virtual rotation axis and to limit lateral movement of the SMP.

### 3.3. Sensor Module Test

**Figure 5:**
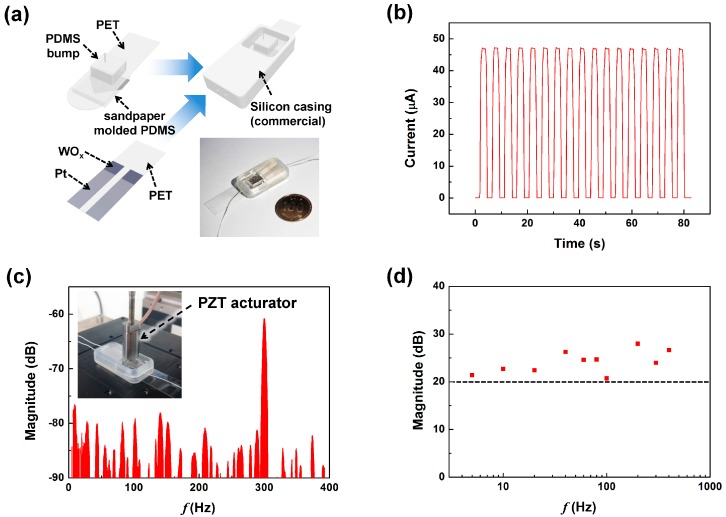
The repeatable pressure sensing operation of dSMP sensor; (**a**) the assembly process of the dSMP sensor, where the inset shows the optical image of the fabricated dSMP sensor; (**b**) the output current of the dSMP sensor during repeated cycles of 1.5 kPa; (**c**) the fast Fourier transform data of the dSMP senor by applying 300 Hz of vibrational pressure, where the inset shows a measurement set-up with PZT acturator, and (**d**) the magnitude of main peak of the dSMP sensor output, which was obtained by subtracting the base line from the main peak, depending on the input vibration frequency between 5~400 Hz, where the black dash line shows 20 dB.

Figure 5a shows the assembly process of the dSMP sensor. For the upper layer, 30 nm of Pt was deposited on the SMP using a stencil mask, then the SMP was attached to a (3-aminopropyl)triethoxylsilane (APTES) treated PET film [23]. A PDMS bump, which acts as a vertical pressure concentrator, was attached to the opposite side of the PET film using the same method. The lower layer has two electrodes in parallel with the WOx making contact to the upper SMP layer. The two layers were placed within a commercial silicon elastic casing that kept the layers placed against each other with minimal pressure to maintain electrical contact and to act as the virtual rotational axis. The inset in Figure 5a shows the optical image of the completely fabricated dSMP sensor. The upper PET film protrudes from a gap placed at the end of the silicon casing, and functions as a lever for the application of external displacement and rotation. Figure 5b shows the output current of the dSMP sensor during repeated cycles of 1.5 kPa vertical pressure application. We observed that the output current variation in the loading state was about 0.53%. Such high stability was a result of the high reliability of the electrical contact between the top SMP electrode and the resistors. PDMS inherently shows a viscoelastic behavior which can cause a slow recovery and results in a hysteresis problem for typical tactile sensor applications [24,25]. Although our sensor was fabricated using PDMS, it showed fast response and recovery times of under 0.3 s. These characteristics were also shown in other reports using metal contacts on PDMS microstructures [18]. The frequency response was investigated by applying vibrational stimulus using a piezoelectric actuator, with 2 μm of free stroke, at the top bump structure, as shown in the inset of Figure 5c. The fast Fourier transform data of the dSMP sensor output by applying 300 Hz of vibrational pressure is shown in Figure 5c. The result shows that, from the sensor measurement data, it was possible to determine the frequency of the applied pressure of 300 Hz. The magnitude fluctuation observed under −80 dB at all frequency regions was assumed to be caused by white noise. Figure 5d shows the magnitude of the sensor output, which was obtained by subtracting the base line from the main peak [26], depending on the input vibration frequency between 5~400 Hz. The dSMP sensor showed a frequency response of over 20 dB at all measured frequencies.

### 3.4. Displacement and Rotaion Angle Sensing Characteristics

**Figure 6:**
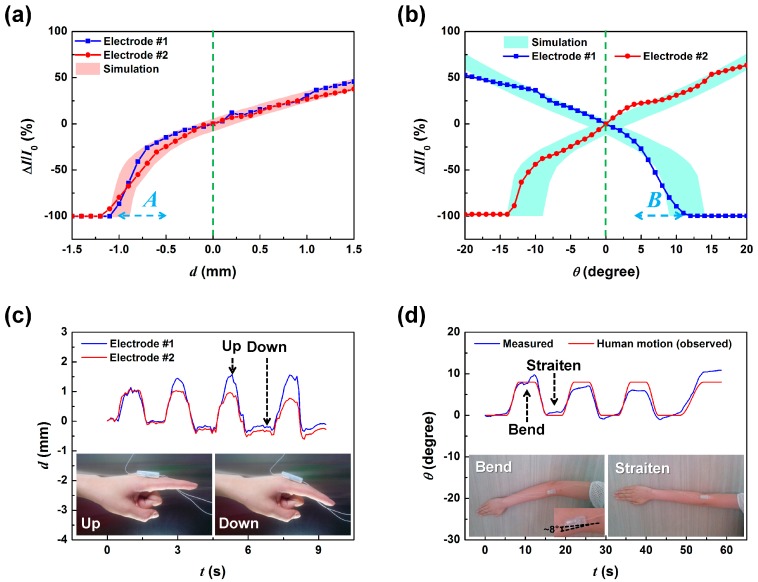
The displacement and rotation angle sensing characteristics of the dSMP sensor; the measured Δ*I*/*I*_0_ of the two lower electrodes depending on applied (**a**) displacement and (**b**) rotation. The pink area represents the simulated output variation, originating from a lateral position error of ±100 μm and a rotation error of ±3 degrees, and the cyan area represents the simulated output variation from an SMP position variation of ±100 μm; (**c**) human forefinger movement sensing result. The insets show the image of the up, and down motion of a human forefinger with the dSMP sensor attached to the proximal phalanx; (**d**) human elbow movement sensing result. The insets show the image of the eight degree bending and straitening motions of a human elbow with the dSMP sensor attached to the side of the elbow joint.

Next, we investigated the displacement and rotation angle sensing characteristics of the dSMP sensor. Figure 6a shows the Δ*I*/*I*_0_ of the two lower electrodes depending on applied displacement. The edge of the SMP triangle was placed 2 mm into the lower electrodes and was centered between them. This was set as the zero position. Displacement was applied by moving the SMP parallel to the lower electrodes that were kept still. We can see that the measured currents of the two electrodes were quite similar with the displacement dependence comparable to the simulated operation. The observed fluctuation of the Δ*I*/*I*_0_ may be caused by the minute vibration of the SMP during displacement sensing, where the simulated output variation originating from a lateral position error of ±100 μm due to vibration was indicated in pink. The friction induced vibrations during the relative motion between the top SMP and the lower electrodes at the contact interface may have caused small variations in contact area resulting in fluctuation in the output current, limiting sensor accuracy. This shows that, although the fluctuation in the output results limited the displacement sensing accuracy to ±33 μm on average ($\bar{A}=\sum \left|I_{m_{i}}-I_{s_{i}}\right|/n$, where $\bar{A}$ is the average accuracy, *I_m_* is the measured current, *I_s_* is the simulated current, and *n* is the number of measurements.), considering that human muscle movement is less acute, the simple dSMP sensor showed adequate displacement sensing functionality [13].

In the displacement range marked as A, in Figure 6a, the SMP and lower electrodes make contact, and the output current begins to increase dramatically at about 15% per 100 μm displacement. Beyond this range, we observed a linear current change of about 2.5% per 100 μm displacement. Figure 6b shows the Δ*I*/*I*_0_ from the two lower electrodes depending on rotation applied to the top SMP layer from −20° to 20°. It can be seen that the dependence of the Δ*I*/*I*_0_ of the two electrodes to the applied rotation were opposite to each other, in contrast to the displacement dependent results. This demonstrates that the dSMP sensor will be able to discriminate between applied displacements from rotation. When rotation was applied clockwise, the output current of electrode no. 1 decreases by 2.7% per degree and in region B, sharply decreases with 12.0% per degree dependence. This was due to the triangular SMP changing contact area as shown in the simulation (Figure 3c). Beyond 12.5 degrees, angular rotation only can be read from the current change in Electrode no. 2. When rotation was applied counter-clockwise from 0, the output current increased by 2.7% per degree in line with simulated results. Therefore, for the rotation sensing operation, observing that rotation has taken place by comparing the current change directions, we may detect the angle for clockwise rotation from the current change measured in electrode no. 2, while for counter clockwise rotation, the current change from electrode no. 1 can be used to measure the rotation angle. The cyan shaded region marks the simulated variation of the rotational angle sensing results when an SMP position variation of ±100 μm was taken into account. The dSMP rotation angle sensing operated within the simulated parameters with an angular sensing accuracy of 0.95 degrees with a ±8.5% average error. The sources of the measurement error may be from the vibrational induced error, observed similarly during displacement sensing, and small fluctuation in the placement of the rotational axis. When initial values are known, both displacement and rotation angle can be determined simultaneously by the change in sensor current values and comparison between different electrode current changes. Using the third electrode, contact pressure change maybe first measured and used to calibrate and determine the displacement. For the calibration, a pre-measured data-set showing pressure dependent displacement current data can be used as a reference. To determine the rotation angle, one only needs to measure the difference in measured current change from the two electrodes to determine the rotation angle, applicable within the measurement angles ±10°. The average of the current changes from the two electrodes can be considered as the displacement measurement with any pressure change applied determined as before. Beyond linear ranges, it would become difficult to determine whether a detected current change was from displacement or rotation since only one of the electrodes would be in contact, making simultaneous measurement impossible.

To test the dSMP sensor in actual multi-axis movement monitoring situation, we attached the sensor on the forefinger and on the elbow and detected the displacement and bending angle upon movement of the joints. Figure 6c shows the displacement sensing results of the dSMP sensor when it was attached to the proximal phalanx of the forefinger (as shown in the inset). The sensor placement allowed it to detect the displacement due to the stretching of the skin layer covering the finger joint. We can see that as the finger was raised and lowered, the dSMP sensor was able to detect the movement. For the first up-and-down motion cycle, we see that the two electrodes detected the same displacement of 1.1 mm. However, upon the following cycles, the measurement results between the two electrodes deviate. We know from basic testing that deviation in the measurements from the two electrodes indicates rotation of the top SMP. Therefore, the deviation shown in the results beyond the first cycle motion must be from the finger actually rotating counterclockwise about 1~3 degrees during the up phase of the motion cycle. This demonstration suggests that our dSMP sensor was able to detect the up-and-down motion of the human finger but also detect, approximately, its rotational angle as well.

To detect the bending and straitening of the human elbow, the dSMP sensor was attached to the side of the elbow and the protruding top PET film was attached to the side of the upper arm as show in the inset of Figure 6d. The measurement results show that the dSMP sensor was able to detect the 8 degree bend-and-straiten cyclic motion of the elbow as angular rotations. The small deviations from the observed bending and the measurement results could be from the displacement of the top SMP, where the bending of the elbow applies compressive strain to the skin around the elbow causing the top SMP on the PET film, attached to the upper arm, to displace inward. While at the last bending cycle, the forearm could have twisted causing the measurement to read as 10 degree bending. These results shows that the ability of the dSMP sensor to detect displacement and rotation angle simultaneously will be a powerful tool for monitoring of human joint movement.

## 4. Triple Electrode SMP Sensor

**Figure 7:**
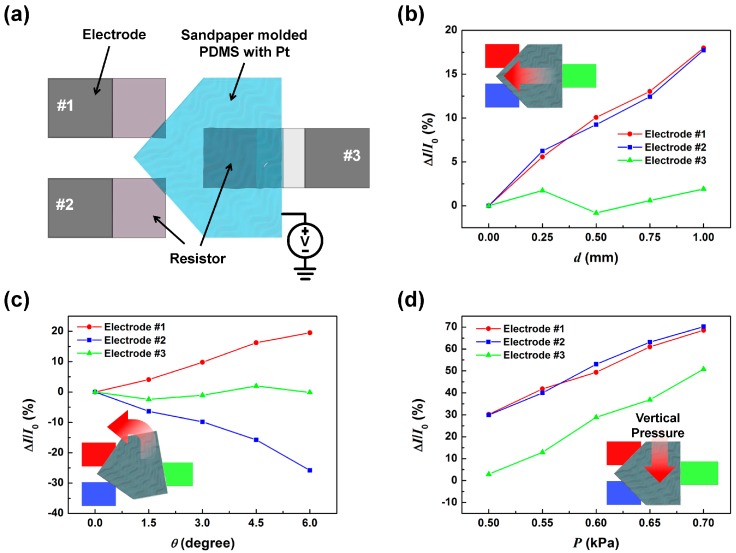
The triple electrode SMP (tSMP) sensor; (**a**) the schematic illustration of the tSMP sensor, and the Δ*I*/*I*_0_ of electrode no. 1 (red), no. 2 (blue) and no. 3 (green) depending on applied displacement (**b**); counter-clockwise rotation (**c**); and vertical pressure (**d**). The insets show the schematic movement illustrations of SMP with color-coded electrodes; no. 1 (red), no. 2 (blue), and no. 3 (green).

As demonstrated, the dSMP sensor was able to detect displacement and rotation angles simultaneously. However, to interpret the measurement data as was done above, it was required that the contact pressure at the SMP and bottom electrode remain the same. For application to realistic situations where external pressure may exist, the applied pressure to the contact area should be considered a variable and be monitored simultaneously as well. As configured, when external pressure is applied to the surface of the dSMP sensor, the result may be interpreted as a positive displacement. This limitation can be overcome by introducing an additional third electrode to the bottom electrode configuration. The schematic illustration of the triple electrode SMP (tSMP) sensor is shown in Figure 7a. An extra resistor on an extra electrode no. 3 were positioned opposite to electrode no. 1 and no. 2, with its resistor centered at the SMP position. The square resistor region was separated from the electrode region to maintain a constant contact area during SMP movement. The resistor only made electrical contact with the external contact area through Pt deposited on the back of the PET strip. For any movement the SMP makes the third electrode would maintain constant contact with this configuration. By measuring the current change at electrode no. 3, it will be possible to detect tactile pressures independently from the other electrodes. It can be seen in Figure 7b,c that for the displacement and rotation angles applied, the third electrode maintained a relatively constant current indicating that no additional pressure was applied to the sensor. When tactile pressure was increased from 0.5 kPA to 0.7 kPa, we can observe from Figure 7d that all of the electrodes showed an increase in current, indicating increased pressure at the contacts. The results demonstrate that the tSMP sensor was able to detect displacement, rotation angle, and tactile pressure simultaneously just by monitoring the change in electrode currents.

## 5. Discussion

As was noted earlier, since the sensor reading depends mostly on the relative positions of the SMP and the bottom electrodes, it becomes essential to develop methods to restrict lateral movement to reduce measurement error, while allowing rotational freedom. For future work, it may be possible to accomplish this by making a slit on the top SMP and place a peg through the slit to the bottom layer which will act as a guide for the SMP movement and an axis of rotation for rotation angle sensing. For actual application to movement monitoring, it could be more advantageous to completely encapsulate the tSMP in an elastomer, which would stabilize the initial position of the SMP relative to the bottom electrodes while also maintaining a minimal contact pressure between the electrodes.

The tSMP sensor provides a simple, low cost method to monitor multi-axis movements. The flexibility and simplicity of the tSMP sensor will give it an advantage over conventional sensors for applications that requires simultaneous measurements of several multi-axis joint movements continuously, such as hand gesture and finger movement monitoring. Also the sensor dimensions can be tailored to meet large displacement and rotation monitoring just by changing the electrode dimensions and relative placements. A reduction in dimension would also be possible for monitoring movements of mechanical elements that are too small for conventional sensors. The tSMP sensor structure may also be applied to multi-functional user input interfaces, such as, a finger-type mouse, a miniature controller, an input button for smart devices, since it will discriminate between pressurized and non-pressurized displacement and rotation allowing a multitude of input levels that cannot be made with current interfaces.

## 6. Conclusions

We have developed a flexible triple contact sensor that was able to simultaneously detect and distinguish between applied displacement, rotation angle, and contact pressure. A sandpaper molded PDMS based top electrode minimized friction at the contact with the bottom resistors, making the measurement non-invasive. The triangular contact area made by the SMP resulted in distinctive change in output current of each contacts making possible displacement, rotation angle, and contact pressure to be monitored simultaneously. The tSMP sensor showed a 0.25 mm^−1^ of displacement sensitivity with a ±33 μm accuracy, a 0.027 degree^−1^ of rotation sensitivity with ~0.95 degree accuracy, and a 4.96 kP^−1^ of pressure sensitivity. For demonstration of possible application to joint movement monitoring, the silicon encased sensor was used to monitor the up-and-down motion of a human forefinger and the bending and straitening motion of a human arm. With further developments, the SMP sensors may provide a low-cost and simple alternative to the current day sensors for all types of joint movement monitoring applications.
